# Respiratory Troubles after an Æsthetics

**Published:** 1902-01-18

**Authors:** 


### RESPIRATORY TROUBLES AFTER ANÆSTHETICS.

At a recent meeting of the Society of Anæsthetics, Mr. H. C. Crouch read a paper by himself and Mr. E. M. Corner, in which he discussed the question whether ether or chloroform was the more likely to be followed by respiratory complications. They had examined every patient who had an anæsthetic in St. Thomas's Hospital during a certain period, and took note of all in whom chest symptoms arose within 24 hours. In all just over 3,000 patients were examined, 2,400 of whom had ether, and 600 chloroform. None of the latter, though they included many mouth cases, had any lung trouble, but ten of the former had, and one of them died. All these ten cases occurred in persons the state of whose lungs was good, and none of them were patients giving a history of having suffered from winter cough. Moreover, they were cases which had not given any trouble to the anæsthetist; they had not been cyanosed, they had not secreted any excessive amount of mucus, nor had they required an unusual amount of ether. In all, however, the operation had lasted a long time. They considered that the after effect was due primarily to the ether, and secondarily to the exposure and tight bandaging after the operation (all but one were trunk cases). It was also a question whether the previous administration of an unduly large quantity of nitrous oxide had not had some share in producing the mischief. They recommend that in all cases which involve tight bandaging, or when the operation is prolonged, chloroform should be the anæsthetic, and that when ether is used a lightly-folded towel should be placed over the patient's mouth until he has been removed to a ward with an equable temperature.

